# The Impact of Treatment with IL-17/IL-23 Inhibitors on Subclinical Atherosclerosis in Patients with Plaque Psoriasis and/or Psoriatic Arthritis: A Systematic Review

**DOI:** 10.3390/biomedicines11020318

**Published:** 2023-01-23

**Authors:** Aikaterini Tsiogka, Stamatios Gregoriou, Alexander Stratigos, Stergios Soulaidopoulos, Natalia Rompoti, Pantelis Panagakis, Marina Papoutsaki, Panagiotis Kostakis, George Kontochristopoulos, Konstantinos Tsioufis, Anna Campanati, Annamaria Offidani, Charalambos Vlachopoulos, Dimitrios Rigopoulos

**Affiliations:** 1 First Department of Dermatology-Venereology, Faculty of Medicine, “A. Sygros” Hospital for Skin and Venereal Diseases, National and Kapodistrian University of Athens, 16121 Athens, Greece; 2 First Cardiology Department, Hippokration General Hospital, School of Medicine, National and Kapodistrian University of Athens, 11527 Athens, Greece; 3 Department of Clinical and Molecular Sciences, Dermatology Clinic, Polytechnic Marche University, 60121 Ancona, Italy

**Keywords:** psoriasis, biologics, IL-23/Th17 axis, cardiovascular, atherosclerosis, arterial stiffness

## Abstract

Accumulating evidence considers psoriasis a systemic inflammatory disorder that is associated with comorbidities such as psoriatic arthritis, cardiovascular disease, and metabolic syndrome. Although the precise pathogenetic links between psoriasis and atherosclerosis warrants further investigation, it is believed that chronic systemic inflammation along with the T helper (Th)-1 and Th17 polarization are associated with endothelial dysfunction and subsequent acceleration of atherosclerosis. Considering the above, several studies have evaluated if optimal control of the inflammation in psoriasis by inhibiting interleukins targeting the Interleukin (IL)-23/Th17 axis could subsequently reduce the atherosclerotic process during anti-psoriatic treatment by using a variety of surrogate markers of subclinical atherosclerosis. This systematic review summarizes current knowledge on the pathogenetic mechanisms and diagnostic evaluation of atherosclerosis in the context of psoriasis and provides a systematic review of the literature on the impact of treatment with biologics targeting the IL-23/Th17 axis on subclinical atherosclerosis in patients with plaque psoriasis and/or psoriatic arthritis.

## 1. Introduction

Psoriasis is a chronic, immune-mediated disease affecting 2–3% of the general population. It is recognized as a systemic inflammatory disease that is associated with comorbidities such as psoriatic arthritis (PsA), cardiovascular disease (CVD), metabolic syndrome, and depression [1]. Except for the increased prevalence of the classical cardiovascular risk factors in patients with psoriasis, including obesity, dyslipidemia, hypertension, and diabetes mellitus, current knowledge considers psoriasis also as an independent risk factor for CVD, as it is thought to increase by 25% the risk for CVD especially in patients with severe skin disease [2,3,4]. This is largely attributed to the chronic systemic inflammation, which leads to endothelial dysfunction and subsequent acceleration of atherosclerosis, as it occurs in other systemic inflammatory diseases, such as rheumatoid arthritis and systemic lupus erythematosus [5,6,7].

Over the recent decades, there have been major advances in the systemic treatment of psoriasis. The introduction of biological agents targeting core inflammatory components of the disease, including tumor necrosis factor-alpha (TNFa) inhibitors, and the interleukin (IL)-12/IL-23 inhibitor ustekinumab, IL-17 and IL-23 inhibitors, has greatly improved the management of psoriasis and, therefore, patients’ quality of life (QoL) [8]. The promising outcomes regarding the efficacy and safety of these agents that has been demonstrated in clinical trials has been confirmed in real-life studies, which have also evaluated their efficacy in sequential use and directly compared different agents of the same category [9,10,11,12]. Literature data suggest that psoriasis and atherosclerosis share common pathogenetic mechanisms and, in this sense, many studies have investigated whether inhibition of the aforementioned inflammatory cytokines to treat psoriasis, could additionally modify the progression of the atherosclerotic process, by evaluating diverse surrogate markers of subclinical atherosclerosis [13,14,15,16,17]. In this regard, the greater amount of evidence exists for TNFa inhibitors, given their longer availability in the market, while data regarding newer biological agents are negligible. As such, many observational and non-controlled studies on TNF-a inhibitors have reported positive findings and suggest that these agents could lead to a reduction of cardiovascular events in patients with psoriasis [18,19,20,21,22,23,24,25,26,27,28]. However, a systematic review of six randomized controlled trials did not provide strong evidence for a positive impact of anti-TNFa agents on subclinical atherosclerosis [29].

The IL-23/T helper-17 (Th17) pathway is recognized to play a fundamental role in psoriasis pathogenesis and is believed to be further implicated in the development of cardiometabolic comorbidities [30,31,32]. The purpose of this article is to summarize current knowledge on the pathogenetic mechanisms and diagnostic evaluation of atherosclerosis in the context of psoriasis and provide a systematic review of the literature on the impact of treatment with biologics targeting the IL-23/Th17 axis on subclinical atherosclerosis in patients with plaque psoriasis and/or PsA.

## 2. Materials & Methods

This systematic review was performed according to the preferred reporting items for systematic reviews and meta-analyses (PRISMA). All research was conducted according to a protocol that was registered in INPLASY (ID number: INPLASY2022120102, DOI: 10.37766/inplasy2022.12.0102). Apart from the systematic review, a narrative review of the literature regarding the diagnostic evaluation of atherosclerosis in the context of psoriasis as well as the common pathogenetic mechanisms between psoriasis and atherosclerosis was performed and is addressed below.

### 2.1. Search Strategy and Eligibility Criteria

The MEDLINE electronic database was searched systematically via PubMed for randomized controlled trials (RCTs) and prospective cohort studies assessing the effect of IL-17 and IL-23 inhibitors on subclinical atherosclerosis in patients with psoriasis from inception to August 2022. Subclinical atherosclerosis could be assessed by any markers or diagnostic examinations, addressed so far in the literature. Non-human studies and articles reporting the effect of IL-17/IL-23 inhibitors on cardiovascular risk factors (e.g., lipid metabolism, adipocytes, etc.) were excluded. Studies reporting the effect of the IL-12/23 inhibitor, ustekinumab, on subclinical atherosclerosis were excluded, as this agent blocks the common p40 subunit of IL-12 and IL-23 and, thus, inhibits not only the IL-23-dependent Th17 but also the IL-12-dependent Th1 immune response. The search strategy is summarized in Table 1.

### 2.2. Selection Process and Data Extraction

The title and abstract review was conducted by two independent reviewers (A.T., S.G.) in order to identify eligible articles. Discrepancies between the two authors that performed the full text review and the quality assessment of the included studies, were performed by the contribution of a third author (D.R.). Reference lists of the included articles were also screened in order to detect any relevant studies that were not identified during the initial search. The following data were extracted by the two independent reviewers (A.T., S.G.): first author, year of publication, type of study, number of participants, type of treatment and follow-up period, assessed marker of subclinical atherosclerosis, and study results.

### 2.3. Study Selection

**Figure 1:**
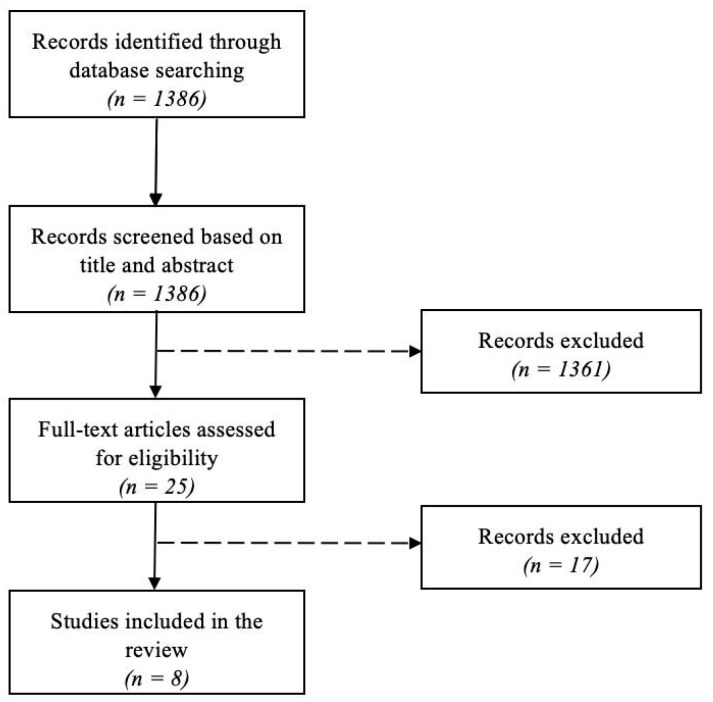
Flow diagram.

The initial search yielded 1386 articles (no filters used). The title and abstract identified 25 articles, which were selected for full text review. Finally, 8 studies were included in the systematic review after the exclusion of irrelevant studies (Figure 1).

## 3. Results of Narrative Review

### 3.1. Diagnostic Evaluation of Subclinical Atherosclerosis in the Context of Psoriasis

To date, many studies have utilized a variety of surrogate markers to evaluate the presence of subclinical atherosclerosis in patients with psoriasis and its evolution during anti-psoriatic treatment, given the broadening understanding of the shared pathophysiology between psoriasis and atherosclerosis. These comprise a wide spectrum of markers, assessed through static or dynamic imaging techniques or blood-based, soluble biomarkers, that either indicate or are indirectly related with early structural or functional alterations of the vasculature, such as endothelial dysfunction (Table 2).

The gold standard method to assess arterial stiffness is carotid-femoral pulse wave velocity (PVW). It is measured by dividing the distance between these two sites with the time taken for a pulse wave to travel between them. PWV is a validated predictor of cardiovascular events and was one of the first markers that was utilized to assess vascular abnormalities in patients with psoriasis compared to healthy controls, supporting that psoriasis is independently associated with increased arterial stiffness [33,34]. The flow-mediated vasodilatation (FMD), which refers to the stimulus-activated (mainly nitric oxide-dependent) vasodilation has been found to be lower in patients with PsA compared to controls [35]. Normally it is performed in the branchial artery, although the radial and the femoral arteries may also be used [36]. Studies have demonstrated that even a 1% increase in FMD is associated with a 13% decrease in the relative cardiovascular risk [37].

The measurement of the intima media thickness (IMT) has also been used to predict coronary artery disease in patients with psoriasis, since thickening of the intima is known to precede the development of plaque and stenosis [38]. High resolution B-mode ultrasonography can be used to measure IMT of the carotid, brachial, or femoral artery, with the latter being more informative [39]. Further static imaging techniques, such as coronary computed tomography angiography (CTA) and fluorodeoxyglucose-positron emission tomography (FDG PET-CT) may be implemented to provide information regarding the coronary plaque characteristics, the epicardial fat thickness, the aortic vascular inflammation, and the coronary calcium score [4,40,41,42,43]. All of the above aid in the prognostication in patients that are at risk of coronary disease and the prediction of cardiovascular outcomes in asymptomatic individuals [44]. Finally, a great variety of soluble biomarkers, including markers of chronic inflammation, markers with pro-atherogenic properties, or endothelial dysfunction markers, have been studied thoroughly, to assess their ability to predict CVD in patients with psoriasis (Table 2) [45,46,47,48,49,50,51,52,53,54,55,56,57,58].

### 3.2. Shared Pathogenetic Mechanisms between Psoriasis and Atherosclerosis

#### 3.2.1. The “Psoriatic March” Concept

**Figure 2:**
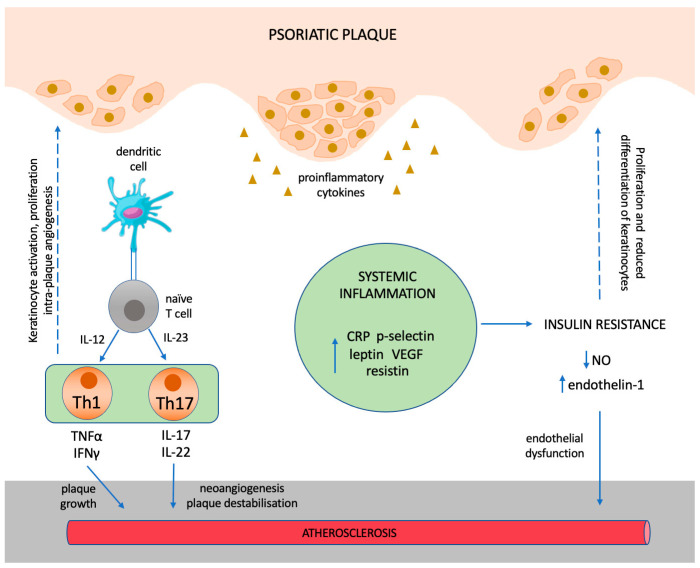
Shared immunologic mechanisms between psoriasis and atherosclerosis.

In 2011, Boehncke et al. proposed a concept of how severe psoriasis may be related to cardiovascular comorbidity. The main principle of the so-called “psoriatic march” is that the increased inflammatory burden of severe psoriasis results in insulin resistance, which, in turn, causes endothelial dysfunction and subsequently atherosclerosis and major adverse cardiovascular events (MACEs) [59]. In particular, it has been observed, that several biomarkers of inflammation, such as C-reactive protein (CRP) and vascular endothelial growth factor (VEGF), as well as adipokines, such as the insulin antagonists resistin and leptin, and indicators of platelet activation, such as P-selectin, are elevated in the blood of psoriatic patients, indicating an increased inflammatory state [46,60,61]. In addition, visceral adipocytes represent a source of proinflammatory mediators, underlining the role of obesity as an aggravating factor for systemic inflammation. The pro-inflammatory cytokines and adipokines could, subsequently, drive insulin resistance, which has been proven to induce endothelial dysfunction, for example by activating the pro-atherogenic mitogen-activated protein kinase (MAPK) pathway in endothelial cells or by inducing nitric oxide (NO)-dependent vasodilatation [62]. As a result, the imbalance between vasodilating and vasoconstricting substances could lead to an abnormal response to physical and chemical stimuli, which characterizes endothelial dysfunction and constitutes an early feature of the atherosclerotic process [63] (Figure 2).

#### 3.2.2. Angiogenesis and Platelet Activation

Angiogenesis constitutes an important inflammatory response in psoriasis, while pro-angiogenic cytokines, including TNFa, IL-8, and IL-17, seem to be involved in the pathogenesis of both psoriasis and atherosclerosis [6]. VEGF, a major angiogenic growth factor, is overexpressed in psoriatic lesions, while this cytokine as well as its receptors have also been found to be expressed in atherosclerotic lesions in coronary arteries [60]. Moreover, diversity in genes which regulate angiogenesis has been linked to increased susceptibility to atherosclerosis [64].

Moreover, the pathophysiology of psoriasis involves platelet activation, a term, which refers to changes in platelet shape, aggregation, and release of platelet components. The assessment of platelet activation may be accomplished with the evaluation of markers, such as the mean platelet volume and expression of the surface antigen, p-selectin (CD62) [65]. The latter serves multiple proinflammatory roles and it has been found to be overexpressed in patients with psoriasis, exhibiting a significant correlation with the Psoriasis Area and Severity Index (PASI) score, and to be further implicated in the development of atherosclerosis [66]. A case-control study of 25 patients with psoriasis and 25 matched healthy individuals identified an increased risk of atherosclerosis, as assessed by increased expression of p-selectin, especially in patients with moderate to severe psoriasis, compared with healthy controls [61].

#### 3.2.3. The Involvement of Th1 and Th17 Immune Responses

Psoriasis is characterized by the Th1 and Th17 polarization of the adaptive immune response, with keratinocytes being activated mainly by the mediators such as interferon gamma (IFN-γ), TNFa, IL-2, and IL-17 [67]. In psoriatic lesions, Th1 cells produce IFN-γ and TNFa, which induce activation and proliferation of keratinocytes as well as the expression of adhesion molecules, whereas Th17 cells secrete IL-17 and IL-22, promoting keratinocyte proliferation and angiogenesis [30]. A recent study of Wang et al. proposed the utilization of IL-17C, the most abundant IL-17 isoform, along with peptidase inhibitor 3 (PI3) as potential biomarkers of effective systemic antipsoriatic treatment end evaluated their relationship to co-existent CVD [68]. Plasma proteins from 36 patients with moderate–severe psoriasis that was effectively treated with either methotrexate or adalimumab or secukinumab or ustekinumab were compared to those from 23 systematically untreated patients. The treated patients exhibited lower levels of IL-17 pathway proteins, while specifically IL-17C and PI3 levels were highly correlated with each other and PASI score. Interestingly, this correlation was modulated in patients with concurrent CVD, who exhibited lower levels of IL-17A compared to those without CVD [68].

The findings of the aforementioned study highlight the complex effects of the IL-17 pathway on psoriasis-related CVD, as IL-17 has been implicated to have both proatherogenic and atheroprotective effects [69]. For instance, low serum IL-17A has been associated with recurrent major cardiovascular events and increased mortality in patients with acute myocardial infarction, suggesting a protective role of IL-17A [70]. On the other hand, a variety of studies demonstrated a reduction of cardiovascular risk in patients with psoriasis that were treated with IL-17 inhibitors (see below). In addition to that, histopathological studies have shown that atherosclerotic plaques contain increased levels of cells IFN-γ, IL-17, and IL-23, with the latter being correlated with disease duration and mortality, implying that both psoriasis and atherosclerosis share common inflammatory cytokine profiles locally and systemically [30]. Differentiated Th1 cells promote plaque growth, while increased levels of intraplaque IL-17 may lead to neoangiogenesis, intensification of inflammation, degradation of the collagen of the fibrous cap, destabilization, and rupture of the atherosclerotic plaque [71,72]. Except for their implication in endothelial dysfunction, the aforementioned cytokines are also considered to be implicated in the pathogenesis of cardiometabolic comorbidities, such as insulin resistance, obesity, and non-alcoholic fatty liver disease [31].

In a study by Gao et al., the production and function of Th17 and Th1 cells was evaluated in atherosclerotic-susceptible mice, where higher expression of IL-17 and retinoic acid-related orphan receptor γt (RORγt) was observed in the arterial wall with plaque than without plaque, and exposure to anti-IL-17 antibodies significantly inhibited the plaque formation [73]. It has to be highlighted that RORC2 is a crucial transcription factor for Th17 cell differentiation. RORγt is expressed in human epidermal keratinocytes and studies have shown disturbed expression in inflammatory skin lesions, including psoriasis, supporting that targeted inhibition of its signaling may represent a promising strategy for the treatment of psoriasis [74,75,76].

## 4. Results of Systematic Search

### Characteristics of Studies Assessing the Impact of Treatment with IL-17 and IL-23 Inhibitors on Subclinical Atherosclerosis in Patients with Psoriasis

According to the results of this systematic review, to date, eight studies have evaluated patients with psoriasis that were treated with biologics targeting the IL-23/Th17 axis, aiming to evaluate their effect on diverse surrogate markers of subclinical atherosclerosis (Table 3).

A prospective, observational study that was conducted by Elnabawi et al. in 2019, assessed the phenotypes of coronary plaques using coronary CTA, in biologic naïve patients that were treated with TNFa-, IL-12/23-, or IL-17- inhibitors compared to those that were treated with topical and/or light therapies, at baseline and after one year [78]. All the participants had low cardiovascular risk by Framingham score (median score, 3) and moderate-to-severe plaque psoriasis (median PASI, 8.6) at baseline. The assessed coronary artery parameters included the total, dense-calcified, and non-calcified plaque burden as well as the plaque morphology index (fibrous, fibro-fatty, and necrotic burden). In one year of treatment, there was a 12% reduction (*p* < 0.001) in non-calcified plaque burden in patients that were receiving anti-IL-17 treatment. Moreover, this group exhibited a significantly greater reduction in coronary plaque burden than that which was observed in the anti-IL-12/23 and non-biologic groups [78].

Changes in the lipid-rich necrotic core (LRNC) during 1-year biologic treatment were assessed in another prospective, observational study of 209 biologic-naïve patients with moderate-to-severe psoriasis [77]. Overall, a statistically significant LRNC reduction [mm^2^; 3.12 (1.99–4.66) versus 2.97 (1.84–4.35) after one year; *p* = 0.028] was observed only in biologic group (TNFa-, IL-12/23-, and IL-17- inhibitors; *n* = 124) and not in those on non-biologic therapy (topical, light, or conventional systemic therapies; *n* = 85). In particular, anti-IL-17 therapy (*n* = 29) reduced the LRNC by 0.39 mm^2^, while no statistically significant LRNC reduction was observed between the different biologic therapy groups [77].

Elnabawi et al. conducted another study to investigate the association of biologic therapy for psoriasis with coronary inflammation using the perivascular fat attenuation index (FAI) via coronary CTA [24]. Overall, 82 patients received biologic therapy with either anti-TNFa, anti-IL-12/23, or anti-IL-17 agents and 52 patients received only topical or light therapy (control group), while most of them had low cardiovascular risk by traditional risk scores and moderate–severe psoriasis. After one year of treatment, there was a significant decrease in FAI compared to baseline only in those that were treated with biologics, with the greater improvement being observed in the anti-IL-17 group [median FAI change (range), −76.92 (−81.16 to −71.67), *p* < 0.001] [24].

In the same year, von Stebut et al. conducted the CARIMA study, a 52-week RCT assessing the endothelial function measured by FMD in patients with moderate–severe psoriasis without evident CVD receiving secukinumab 300 mg vs. 150 mg for 52 weeks or placebo until week 12, followed by secukinumab 300 mg vs. 150 mg until week 52 [83]. Although no statistically significant change was observed at week 12, the mean FMD increased across the groups until week 52 and it was significantly higher compared to baseline in patients receiving secukinumab 300 mg continuously for 52 weeks [+2.1%, 95% confidence interval (CI) 0.8–3.3; *p* = 0.0022]. Secondary endpoints, including PWV, augmentation index, blood-based biomarkers, and plaque burden by MRI, did not exhibit relevant changes during the study [83].

In a following cohort study by Makavos et al., anti-IL-17 treatment was compared to conventional treatment with cyclosporine or methotrexate regarding their ability to improve myocardial deformation and vascular function in moderate–severe psoriasis [80]. A total of 150 patients received either secukinumab (*n* = 50), cyclosporine (*n* = 50), or methotrexate (*n* = 50) for 12 months. At the end of treatment, anti-IL-17 treatment led to greater improvement of myocardial and vascular function, as assessed by global longitudinal strain, left ventricular twisting/untwisting indices, coronary flow reserve (CFR) (CFR change at 12 months: 19%, *p* = 0.02), and PWV (PWV reduction at 12 months: −11%, *p* = 0.04). Interestingly, higher PWV values were observed in the cyclosporine group (PWV increase at 12 months: 14%). Soluble markers of oxidative stress (malondialdehyde, protein carbonyl) were reduced only in the anti-IL-17 group (*p* < 0.05) [80].

Piros et al. conducted another observational study on 31 severe psoriatic patients who received secukinumab (*n* = 20) or ixekizumab (*n* = 11), in order to evaluate the impact of biologic therapy on vascular wall inflammation, as assessed by arterial IMT after 6 months of treatment [82]. All of the indices, including right and left carotid, right and left branchial as well, as right and left femoral IMT, exhibited a statistically significant reduction compared to baseline [82].

In contrast to the aforementioned positive findings, the two following studies failed to demonstrate a significant improvement of the selected parameters of subclinical atherosclerosis in patients with psoriasis. Marovt et al. evaluated 15 biologic-naïve patients, who received either ustekinumab (*n* = 4), secukinumab (*n* = 10), or ixekizumab (*n* = 1) for 6 months [81]. At the end of treatment, no significant changes in the mean (left and right carotid) IMT (*p* = 0.737) and carotid-femoral PWV (*p* = 0.031) were observed, although it has to be highlighted that a short observational time of six months was determined [81]. A placebo-controlled RCT (VIP-S) that was conducted by Gelfand et al. sought to assess the effect of secukinumab therapy on aortic vascular inflammation using FDG-PET/CT and other soluble biomarkers during 52 weeks of treatment in patients with moderate–severe psoriasis [84]. Initially, 46 patients were randomized to secukinumab and 45 to the placebo group during the first 12 week placebo-controlled period, from whom 86 entered the following 40 week treatment period with secukinumab. At week 12, there was no statistically significant difference in aortic inflammation between the secukinumab and placebo groups. A neutral effect of secukinumab on vascular inflammation and cardiometabolic biomarkers (e.g., lipoprotein, adiposity, insulin resistance) was also observed at the end of treatment [79].

## 5. Discussion and Future Perspectives

Although the precise pathogenetic links between psoriasis and atherosclerosis warrants further investigation, it is increasingly recognized that inflammatory processes involving the Th1 and Th17 immune responses have a prominent role in their shared pathogenesis as well as in the pathogenesis of the most cardiometabolic psoriasis-associated conditions. To date, some studies have evaluated if optimal control of the inflammation in psoriasis by inhibiting interleukins targeting the IL-23/Th17 axis could subsequently reduce the accompanied atherosclerotic process, providing conflicting results. In particular, this systematic review has identified six observational studies and two RCTs that evaluated the effect of anti-IL-17 therapy on diverse surrogate markers of subclinical atherosclerosis. A total of five of them utilized different static imaging techniques to evaluate structural changes of the vasculature, and two utilized dynamic imaging techniques to assess arterial stiffness. Although six studies demonstrated positive effects of biologic therapy, two showed no statistically significant benefit on subclinical atherosclerosis. Interestingly, during the literature search, no studies assessing the effect of IL-23 inhibitors were identified.

Some further studies assessed the effect of biological agents on psoriasis-associated CVD and cardiometabolic comorbidities, including diabetes, lipoprotein function, obesity/adipokines, and other metabolic parameters [84,85,86,87,88]. A systematic review and meta-analysis, that was conducted by Gonzáles-Cantero et al., included five RCTs, that examined the impact of adalimumab, ustekinumab, or secukinumab on imaging (aortic vascular inflammation on PET-CT, FMD) and biomarkers of CVD (lipoproteins, inflammation, obesity, insulin resistance) [14]. It has been shown that ustekinumab reduced aortic vascular inflammation and TNF-a inhibitors reduced CRP and IL-6. Otherwise, there was no beneficial effect on the assessed biomarkers in patients that were receiving biologics compared to those that were exposed to the placebo [14]. Interestingly, a meta-analysis of RCTs that was published in 2011, reported a potential risk of severe cardiovascular events during the initiation period of ustekinumab [89]. This observation was supported by a recent case-control study (*n* = 9290), which suggested that the initiation of ustekinumab may trigger severe cardiovascular events in patients presenting with high cardiovascular risk [90]. Finally, a recent systematic review and meta-analysis on vascular inflammation (PET-CT) and its evolution during treatment with biologics underlined the association between psoriasis and aortic vascular inflammation but it could not support a beneficial effect of biologic treatment [91].

Considering the growing amount of evidence supporting the systemic inflammatory nature of psoriasis and its association with an increased prevalence of cardiometabolic comorbidities, the last EuroGuiderm guideline on the treatment of psoriasis vulgaris, published in 2020, provided detailed recommendations regarding the management of patients with comorbid situations, including ischemic heart disease/atherosclerosis. In particular, appropriate investigations and treatment should be initiated in accordance with current European Society of Cardiology guidance in patients with established CVD, while regarding antipsoriatic treatment, the authors suggested the use of methotrexate, TNF-a inhibitors, ustekinumab, and IL-17 inhibitors and avoidance of cyclosporine or acitretin in patients with psoriasis and ischemic heart disease [92,93].

The main limitation of the present review is the heterogeneity of the included studies, especially regarding the diagnostic methods that were utilized to assess the impact of biologics on subclinical atherosclerosis in psoriasis patients, prohibiting a meta-analysis of outcomes and subgroup analysis, to draw stronger conclusions on this topic. Another limitation is that only the PubMed search engine was used for study selection, which provides access in most studies with high level of evidence, such as RCTs and prospective cohort studies, which were included in the present study.

Considering the above, it is yet to be elucidated if the aforementioned discrepancies on the anti-atherogenic effect are associated with differences in patient-related characteristics, principally with regards to their CVD-risk profile at the onset of biologic therapy, drug-related characteristics such as pharmacologic properties of each IL-17 inhibitor, or difference in sensitivity between the utilized diagnostic techniques. In the era of the increasing understanding of the involvement of systemic inflammation in the pathogenesis of atherosclerosis and consideration of psoriasis as an independent cardiovascular risk factor, future research could include the identification of clinical markers in patients with psoriasis and/or PsA that could predict an optimal anti-atherogenic effect of systemic treatment of psoriasis with biologics. In that respect, an important aspect could be the establishment of sensitive but also accessible and cost-effective diagnostic methods to be included in the evaluation of the cardiovascular status in patients with moderate–severe psoriasis, especially in those presenting with additional classical cardiovascular risk factors. Finally, future, well-controlled studies could elucidate if different biologic agents may exhibit a more pronounced anti-atherogenic effect and target CVD inflammation more effectively, an association which is yet particularly to be studied in patients receiving IL-23 inhibitors, considering the sparsity of such data in the medical literature.

## Figures and Tables

**Table 1:** MEDLINE search strategy *.

Search Terms	Results
Cardiovascular	2,051,829
Coronary disease	355,309
Atherosclerosis	165,432
Cardiovascular OR coronary disease OR atherosclerosis	2,286,796
Psoriasis	59,808
Psoriatic arthritis	12,928
psoriasis OR psoriatic arthritis	62,289
Biologics	7,336,51
Interleukin 17 inhibitor	2846
Interleukin 23 inhibitor	1077
Secukinumab	1734
Brodalumab	473
Ixekizumab	875
Guselkumab	473
Rizankizumab	296
Biologics OR interleukin 17 inhibitor OR interleukin 23 inhibitor OR secukinumab OR brodalumab OR ixekizumab OR guselkumab OR rizankizumab	7,337,887
(cardiovascular OR atherosclerosis OR coronary disease) AND (psoriasis OR psoriatic arthritis) AND (biologics OR interleukin 17 inhibitor OR interleukin 23 inhibitor OR secukinumab OR brodalumab OR ixekizumab OR guselkumab OR rizankizumab)	1386

* via PubMed, performed on 31 August 2022, no filters used.

**Table 2:** Surrogate markers of subclinical atherosclerosis in patients with psoriasis.

Marker	Diagnostic Method	Comments
Assessment of functional alterations		
Pulse wave velocity	High resolution B-mode ultrasound	Gold standard measurement of arterial stiffness
Flow-mediated dilatation	High resolution B-mode ultrasound	assesses stimulus-activated (mainly nitric oxide-dependent) vasodilation, normally performed in the brachial artery, some studies have also used the radial and femoral arteries
Assessment of structural alterations		
Intima media thickness	High resolution B-mode ultrasound	-carotid, branchial, femoral (more informative), thickening of the intima precedes the development of plaque and stenosis
Coronary artery calcium (CAC score)	Non-contrast coronary artery calcium CT	Measures the amount of calcium in coronary arteries, indicates cardiovascular disease, assists in cardiovascular risk assessment
Coronary plaque characterization	Coronary CTA	e.g., total coronary plaque burden, non-calcified coronary plaque burden, high risk plaque prevalence
Lipid-rich necrotic core	Coronary CTA	High risk coronary plaque feature, histopathologic correlate of low-attenuation plaque
Perivascular fat attenuation index	Coronary CTA	Quantification of coronary inflammation, may predict the risk of developing atherosclerosis
Aortic vascular inflammation	FDG PET scan	Marker of subclinical vascular disease, predictive of future major cardiovascular events
Epicardial fat thickness	Native CT, MRI, TTE	Epicardial adipose tissue functions as a lipid store that secrets hormones/cytokines etc., may be related with disease duration
Soluble biomarkers		
N-terminal pro B-type natriuretic peptide (NT-proBNP), homocysteine, sCD40L, soluble lectin-like oxidized low-density lipoprotein receptor-1 (sLOX-1), leptin, high sensitivity C-reactive protein (hs-CRP), fetuin-A, cystatin-C, osteopontin, chemerin, GlycA, endocan, vascular endothelial growth factor (VEGF), YKL-40, leptin, fetuin-A, cystatin-C, psoriasin, koebnerisin		

CT, computed tomography; CTA, computed tomography angiography; FDG PET-CT, fluorodeoxyglucose-positron emission tomography; MRI, magnetic resonance imaging; NMR, nuclear magnetic resonance; TTE, transthoracic echocardiography.

**Table 3:** Included studies, listed alphabetically based on first author’s name.

First Author, Year	Study Type	*n*	Intervention	Follow Up	Assessed Marker of Subclinical Atherosclerosis	Results
Choi et al., 2020 [77]	Prospective, cohort	209	Group A (*n* = 124): biologic therapy (anti-TNFa, anti-IL12/23, anti-IL-17) Group B (*n* = 85): non biologic therapy (topical, light, systemic therapy)	1 year	Lipid-rich necrotic core assessed by CTA	-Favorable modification of lipid-rich necrotic core in patients under biologics -No significant difference between different biologic groups
Elnabawi et al., 2019 [24]	Prospective, cohort	134	Group A (*n* = 82): anti-TNFα, anti-IL-12/23, anti-IL17) Group B (*n* = 52): non biologic therapy (topical, light therapy)	1 year	Perivascular fat attenuation index assessed by coronary CTA	-Significant decrease in median fat attenuation index only in biologic group -Similar changes between different biologic groups
Elnabawi et al., 2019 [78]	Prospective, cohort	121	Group A (*n* = 89): biologic therapy (anti-TNFa, anti-IL-12/23, anti-IL-17) Group B (*n* = 32): non biologic therapy (topical/light therapy)	1 year	Coronary plaque burden and plaque subcomponents (calcified vs. non-calcified) assessed by coronary CTA	Favorable modulation of coronary plaque indices
Gelfand et al., 2020 (VIP-S) [79]	RCT	91	12-week period [Secukinumab (*n* = 46) vs. placebo (*n* = 45)] followed by a 40-week period [secukinumab (*n* = 86)]	1 year	Aortic vascular inflammation assessed by FDG-PET/CT	Non-statistically significant −0.75% reduction in target-to-blood at week 12 and at week 52
Makavos et al., 2020 [80]	Prospective, cohort	150	Secukinumab (*n* = 50) vs. cyclosporine (*n* = 50) vs. methotrexate (*n* = 50)	1 year	GLS, GLSR, GLSRE, LVtwist and untwisting, CFR, PWV, MDA, PC	Greater improvement of all markers in secukinumab group
Marovt et al., 2020 [81]	Prospective, cohort	15	Ustekinumab (*n* = 4) vs. secukinumab (*n* = 10) vs. ixekizumab (*n* = 1)	6 months	PWV, IMT	No significant changes in all groups
Piros et al., 2021 [82]	Prospective, cohort	31	Secukinumab (*n* = 20) vs. ixekizumab (*n* = 11)	6 months	IMT	Significant reduction of IMT
von Stebut et al., 2019 (CARIMA) [83]	RCT	151	Secukinumab 300 mg for 52 weeks (*n* = 48) vs. secukinumab 150 mg for 52 weeks (*n* = 54) vs. placebo for 12 weeks followed by secukinumab 300 mg for 40 weeks (*n* = 26) vs. placebo for 12 weeks followed by secukinumab 150 mg for 40 weeks (*n* = 23)	1 year	FMD	Non-significant difference in FMD until week 12; significantly improved FMD in patients receiving secukinumab 300 mg for 52 weeks

CFR, coronary flow reserve; CTA, computed tomography angiography; FDG-PET/CT, fluorodeoxyglucose-positron emission tomography/computed tomography; FMD, flow-mediated dilatation; GLS, global longitudinal strain; GLSR, global longitudinal strain rate; GLSRE, global longitudinal strain rate at early diastole; IL, interleukin; IMT, intima media thickness; LV, left ventricular; MDA, malondialdehyde; PC, protein carbonyl; PWV, pulse wave velocity; RCT, randomized controlled trial; TNFa, tumor necrosis factor alpha.

## Data Availability

The authors confirm that the data supporting the findings of this study are available within the article.
